# Research Progress on Inflammation and Immune Dysregulation in PTSD

**DOI:** 10.1002/brb3.70633

**Published:** 2025-06-17

**Authors:** Luodong Yang, Jiaying Lu, Zhiqiang Zhao, Ziwei Zhang, Weiliang Yang, Guiqing Zhang

**Affiliations:** ^1^ The First Affiliated Hospital of Shihezi University Shihezi China; ^2^ The Clinical Medical Research Center for Mental and Psychological Disorders of the Xinjiang Production and Construction Corps Shihezi China; ^3^ The Sub‐Center of the National Clinical Medical Research Center for Mental and Psychological Disorders Xinjiang Production and Construction Corps Shihezi China

**Keywords:** immune cells, immune genes, inflammatory cytokines, pathological mechanisms, post‐traumatic stress disorder

## Abstract

**Introduction:**

Post‐traumatic stress disorder (PTSD) is a severe psychological condition triggered by traumatic events, commonly characterized by symptoms such as re‐experiencing traumatic memories, avoidance, hyperarousal, and disturbances in cognition and emotions. While PTSD is often viewed through a psychological lens, increasing evidence highlights its strong association with immune system dysfunction and inflammation. This narrative review summarizes recent research progress on the role of inflammation and immune dysregulation in PTSD, highlighting key findings and their implications for understanding the pathophysiology of the disorder.

**Methods:**

We conducted a search in the PubMed and Web of Science databases using the keywords “PTSD” and “related inflammatory markers” and discussed the existing literature on the relationship between PTSD and inflammatory responses.

**Results:**

The research indicates that PTSD is marked by significant imbalances in pro‐inflammatory and anti‐inflammatory cytokines across various biological fluids, including blood, saliva, and cerebrospinal fluid. Abnormal immune cell activation and elevated levels of soluble adhesion molecules, chemokines, and markers of inflammation were frequently observed in PTSD patients. These inflammatory responses are accompanied by aberrant activity in central immune cells, suggesting that inflammation may play a key role in the pathogenesis of PTSD. In addition, neuroinflammatory processes were linked to cognitive and emotional disturbances commonly seen in individuals with PTSD.

**Conclusion:**

Our findings suggest that immune system dysfunction and inflammation are integral components of PTSD pathology. Understanding the mechanisms of neuroinflammation and immune dysregulation could facilitate the early identification of individuals at high risk for PTSD and pave the way for inflammation‐targeted therapies. Future research should focus on developing novel anti‐inflammatory interventions to complement existing therapeutic approaches, potentially offering new avenues for precision treatment strategies for PTSD.

## Graphical abstract

Post‐traumatic stress disorder (PTSD) is associated with a complex interplay between immune dysregulation and inflammatory responses. Microglia and astrocytes in the central nervous system play a key role in the immune response in PTSD, while elevated pro‐inflammatory cytokines in the peripheral system further exacerbate inflammation. In addition, peripheral immune cells that cross the blood–brain barrier (BBB) into the brain may intensify the inflammatory response in the central nervous system. The pathogenesis of PTSD is closely linked to autonomic dysfunction, dysregulation of the hypothalamic‐pituitary‐adrenal (HPA) axis, epigenetic changes, and sterile inflammation. In the future, anti‐inflammatory therapies may emerge as a potential treatment strategy for PTSD, offering new avenues for clinical intervention.

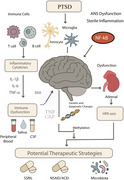

## 1 Introduction

Post‐traumatic stress disorder (PTSD) is a severe and debilitating mental disorder triggered by traumatic events. The core clinical manifestations include recurrent re‐experiencing of the traumatic event, avoidance of related cues, negative alterations in mood and cognition, and symptoms of hyperarousal. According to the *Diagnostic and Statistical Manual of Mental Disorders* (Fifth Edition) (DSM‐5), PTSD is classified as an axis I disorder, with diagnostic criteria requiring the persistence of these symptoms for at least 1 month, causing significant impairment in daily functioning and quality of life (Florido et al. 2023). Global surveys reveal that approximately 70% of individuals experience at least one traumatic event in their lifetime, with 30.5% having encountered four or more traumatic events, such as natural disasters, wars, life‐threatening illnesses, or major accidents (Benjet et al. 2016). However, not all individuals exposed to traumatic events develop PTSD. The risk of its onset is influenced by a combination of factors, including individual characteristics, the type of trauma, the age at which it occurred, and the frequency of exposure.

PTSD not only severely impairs mental health but also has profound impacts on physical health and social functioning. Studies have shown that individuals with PTSD have significantly higher utilization of healthcare resources compared to the general population, leading to increased medical expenditures (Rieka et al. 2020). In addition, PTSD is closely associated with various severe mental and physical illnesses, including major depressive disorder (Marra et al. 2024), asthma (Wisnivesky et al. 2024), cardiovascular diseases (Seligowski et al. 2022), and autoimmune disorders (Zeng et al. 2023). More concerning is the significant association between PTSD and an increased incidence of Parkinson's disease (Prieto et al. 2023) as well as a heightened risk of self‐harm (Sala‐Hamrick et al. 2023). In recent years, longitudinal studies have revealed a close link between PTSD and various autoimmune diseases, including endocrine disorders, inflammatory arthritis, vasculitis, and diseases affecting the nervous, digestive, and connective tissue systems (Maihofer et al. 2024). This series of associations suggests that the pathological mechanisms of PTSD may extend beyond the traditional psychological domain, involving widespread systemic physiological dysregulation, particularly abnormalities in immune system function.

An increasing body of evidence indicates that patients with PTSD exhibit significant features of inflammatory activation and immune dysregulation (Biltz et al. 2022; Núñez‐Rios et al. 2022). These manifestations primarily include elevated levels of pro‐inflammatory cytokines in the peripheral blood and central nervous system (Chan et al. 2023), excessive activation of immune cells (Li et al. 2021; Valenza et al. 2024; L. L. Zhang et al. 2024), and disrupted neuroimmune interactions (Chan et al. 2023; Guo et al. 2023; L. L. Zhang et al. 2024). Furthermore, inflammatory responses may contribute to alterations in brain structure and function (Y.‐K. Kim et al. 2019; Mehta et al. 2022), thereby exerting profound effects on learning, memory, and emotional regulation, which exacerbate PTSD symptoms (Mehta et al. 2020; Moodley et al. 2023). This “neuroinflammation hypothesis” has been progressively validated in recent foundational research (Table 1).

**TABLE 1:** Alterations in cytokines observed in patients with PTSD.

Signal	Sample source	Increased	Decreased	Negative
IL‐1β	Blood	Koirala et al. (2023), Tanaka et al. (2021), von Känel et al. (2007);von Känel et al. (2022), Wang et al. (2019), Y. Zhang et al. (2023)	—	—
IL‐2	Blood	Fonkoue et al. (2023), J.‐J. Yang and Jiang (2020)	—	Tursich et al. (2014)
	Saliva	Wang et al. (2016)	—	—
IL‐4	Blood	—	—	von Känel et al. (2007)
	Saliva	—	Wang et al. (2016)	—
IL‐6	Blood	Hori et al. (2020), Koirala et al. (2023), Malik et al. (2022), Maihofer et al. (2024), Rhein et al. (2021), Tanaka et al. (2021), Toft et al. (2022), von Känel et al. (2022), Y. Zhang et al. (2023)	—	von Känel et al. (2007)
	Saliva	Wang et al. (2016)	—	—
	Cerebrospinal fluid	Kim et al. (2020)	—	—
IL‐10	Blood	Malik et al. (2022), Renner et al. (2022), Toft et al. (2022)	—	von Känel et al. (2007)
	Saliva	—	Wang et al. (2016)	—
IL‐17	Blood	Zuo et al. (2023)	—	—
	Saliva	Wang et al. (2016)	—	—
IFN‐γ	Blood	Lindqvist et al. (2017), Tanaka et al. (2021), J.‐J. Yang and Jiang (2020)	—	Michopoulos et al. (2020)
	Saliva	Wang et al. (2016)	—	—
TNF‐α	Blood	Malik et al. (2022), Tanaka et al. (2021), von Känel et al. (2007), von Känel et al. (2022), Wang et al. (2019), Y. Zhang et al. (2023)	—	—
CRP	Blood	Malik et al. (2022), Maihofer et al. (2024), Plantinga et al. (2013), Y. Zhang et al. (2023)	—	von Känel et al. (2007)
	Saliva	An et al. (2015), Robles et al. (2024)	—	—
hs‐CRP	Blood	Otsuka et al. (2021), Plantinga et al. (2013), Sumner et al. (2017)	—	—
ICMA‐1	Blood	Plantinga et al. (2013), Sumner et al. (2017)	—	—

Given the widespread negative impact of PTSD on individuals and society, it is crucial to thoroughly investigate its pathogenesis, particularly in the area of inflammation‐related mechanisms. Such research not only deepens our understanding of the biological underpinnings of PTSD but also offers new avenues for early screening and intervention. Furthermore, studies on the inflammatory mechanisms of PTSD provide theoretical support for developing precision therapeutic strategies based on immune modulation. In the future, deeper exploration in this field will contribute to more comprehensive and effective diagnostic and treatment approaches for PTSD patients.

## 2 Inflammation and PTSD

### 2.1 Activation of Cytokines

Cytokines are a class of secreted signaling proteins that play a crucial role in regulating immune responses, including the modulation of immune defense against pathogens and inflammatory responses to tissue damage (Jiang et al. 2021). They are primarily secreted by peripheral immune cells (such as macrophages, lymphocytes, and mast cells), vascular endothelial cells, and immune cells within the central nervous system (such as microglia, astrocytes, and neurons) (Kaminska et al. 2024). Based on their functions, cytokines are categorized into pro‐inflammatory and anti‐inflammatory types, which interact to maintain a dynamic balance in the regulation of inflammation.

#### 2.1.1 Imbalance of Cytokine Levels in PTSD Patients

In the study of PTSD, the relationship between cytokines and inflammatory responses has become a focal point. Numerous studies have found significantly elevated levels of pro‐inflammatory cytokines in individuals with PTSD, such as interleukin‐1β (IL‐1β) (Koirala et al. 2023; Tanaka et al. 2021; von Känel et al. 2007; von Känel et al. 2022; W. Wang et al. 2019; Y. Zhang et al. 2023), interleukin‐2 (IL‐2) (Fonkoue et al. 2023; J.‐J. Yang and Jiang 2020), interleukin‐6 (IL‐6) (Hori et al. 2020; Koirala et al. 2023; Malik et al. 2022; Maihofer et al. 2024; Rhein et al. 2021; Tanaka et al. 2021; Toft et al. 2022; von Känel et al. 2022; Y. Zhang et al. 2023), interferon‐γ (IFN‐γ) (Lindqvist et al. 2017; Tanaka et al. 2021; J.‐J. Yang and Jiang 2020), tumor necrosis factor‐α (TNF‐α) (Malik et al. 2022; Tanaka et al. 2021; von Känel et al. 2007; von Känel et al. 2022; W. Wang et al. 2019; Y. Zhang et al. 2023), and C‐reactive protein (CRP) (Malik et al. 2022; Maihofer et al. 2024; Plantinga et al. 2013; Y. Zhang et al. 2023). In addition, elevated levels of high‐sensitivity C‐reactive protein (hs‐CRP), intercellular adhesion molecule‐1 (ICAM‐1), and chemokines have been widely reported in PTSD patients (Otsuka et al. 2021; Plantinga et al. 2013; Sumner et al. 2017; L. Zhang et al. 2020). Studies have further revealed that these cytokine and soluble receptor levels are closely associated with structural and functional alterations in certain brain regions, such as the hippocampus and prefrontal cortex (Mehta et al. 2022; Mehta et al. 2020). A study has also found that the NF‐κB pathway activity is enhanced in women with PTSD who have experienced child abuse, and this enhancement is positively correlated with the severity of symptoms (Pace et al. 2012), further supporting the central role of inflammation in the pathogenesis of PTSD.

Although the majority of research findings are consistent, some studies have reported contradictory results. For example, one study found no significant changes in the levels of IL‐2, IL‐4, IL‐6, IL‐10, IFN‐γ, and CRP in the peripheral blood of PTSD patients compared to the control group (Michopoulos et al. 2020;Tursich et al. 2014; von Känel et al. 2007), with an anomalous increase in the anti‐inflammatory cytokine IL‐10 levels specifically in female patients (Malik et al. 2022; Renner et al. 2022; Toft et al. 2022). In addition, Mendelian randomization analysis revealed that genetically predicted elevated IL‐17 levels might be associated with a lower risk of PTSD (Zuo et al. 2023). These discrepancies suggest that individual differences, sample characteristics, and research methodologies should be carefully considered when investigating the relationship between PTSD and cytokines.

#### 2.1.2 Alterations in Saliva and Cerebrospinal Fluid in PTSD Patients

**FIGURE 1:**
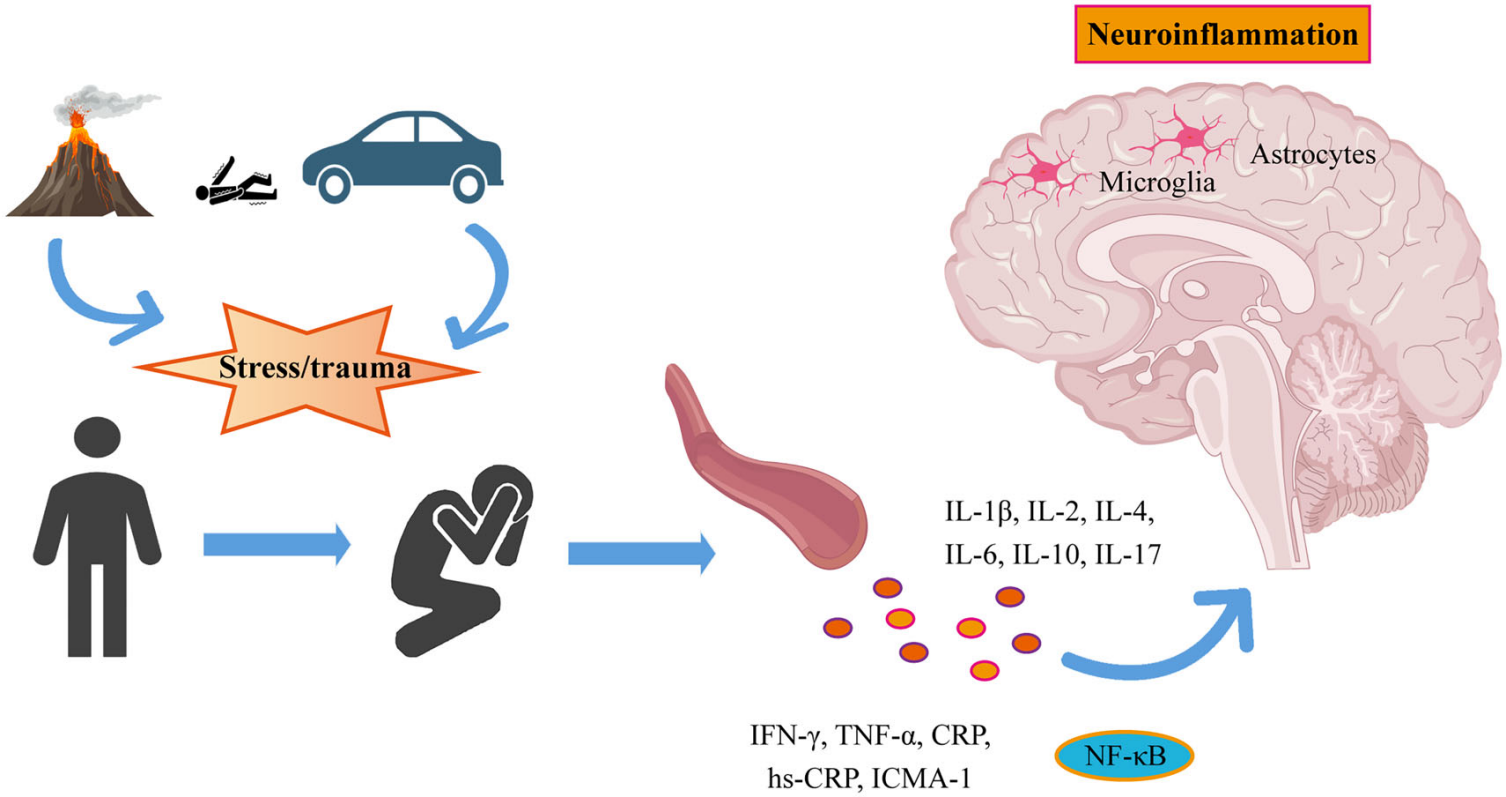
The immune cells and cytokines associated with PTSD.

The use of saliva as a non‐invasive sample has further advanced PTSD‐related research. Studies have shown that in veterans, salivary levels of pro‐inflammatory cytokines (such as IL‐2, IFN‐γ, IL‐6, and IL‐17) are elevated, while anti‐inflammatory cytokine levels (such as IL‐4 and IL‐10) are reduced, with these changes being even more pronounced than those observed in blood samples (Z. Wang et al. 2016). In addition, salivary levels of CRP and chemokines are significantly associated with PTSD symptoms (An et al. 2015; Robles et al. 2024) (Figure 1). Moreover, cerebrospinal fluid (CSF) analysis has further confirmed the inflammatory state in PTSD patients, particularly with significantly elevated levels of IL‐6 in the CSF (B. K. Kim et al. 2020).

#### 2.1.3 Alterations in Animal Models

Animal model studies provide mechanistic support for understanding the complex relationship between PTSD and cytokines (Table 2). These studies have shown that stress not only induces elevated levels of peripheral pro‐inflammatory cytokines (such as IL‐1β and IL‐6) but also activates inflammatory pathways in specific regions of the central nervous system, such as the hippocampus and amygdala, characterized by significantly increased Caspase‐1 activity and NLRP3 inflammasome activation (Dong et al. 2020; Ghosh et al. 2021; L. Yang et al. 2024). Similar studies have reported significantly elevated levels of IL‐1β, IFN‐γ, TNF‐α, and nitric oxide (NO) in the prefrontal cortex and hippocampus, further supporting the hypothesis that excessive activation of inflammatory pathways may be one of the key mechanisms underlying the pathogenesis of PTSD (S.‐C. Wang, Lin, et al. 2018; M. Wang, Duan, et al. 2018).

**TABLE 2:** Changes in cytokines observed in animal models of PTSD.

Signal	Location	Increased	Decreased	Negative
IL‐6	Hippocampus and amygdale	Ghosh et al. (2021)	—	—
IL‐1β	Hippocampus and amygdale	Ghosh et al. (2021), Wang et al. (2018)	—	—
	Hippocampus	Dong et al. (2020), Wang et al. (2018), L. Yang et al. (2024)	—	—
IFN‐γ	Medial prefrontal cortex; hippocampus and amygdale	Wang et al. (2018)	—	—
	Hippocampus	Wang et al. (2018)	—	—
TNF‐α	Medial prefrontal cortex; hippocampus and amygdale	Wang et al. (2018)	—	—
	Hippocampus	Wang et al. (2018)	—	—

It is worth noting that the roles of pro‐inflammatory and anti‐inflammatory cytokines are not limited to the periphery; they may also act by influencing central inflammatory pathways. For instance, peripheral cytokines can cross the blood–brain barrier and act on microglial cells, triggering their activation and leading to the release of additional pro‐inflammatory cytokines, thereby exacerbating neuroinflammation (Karavelioğlu et al. 2014). This mechanism highlights the complex interactions between peripheral and central inflammation, providing important insights into the pathogenesis of PTSD.

### 2.2 Activation of Immune Cells

The relationship between PTSD and immune cells encompasses the complex interactions of the innate immune system (such as monocytes, macrophages, dendritic cells, and microglial cells) and the adaptive immune system (such as T lymphocytes and B lymphocytes) (Katrinli et al. 2022).

#### 2.2.1 The Role of Central Immune Cells

Microglia, as specialized immune cells in the brain, account for 5%–10% of total brain cells. They not only possess phagocytic functions similar to macrophages but also play a critical role in neural regulation. Under stress or pressure stimuli, microglia become activated and release large amounts of pro‐inflammatory cytokines, inducing astrocytes to secrete additional inflammatory factors. This creates a positive feedback loop, triggering and sustaining the inflammatory state of the central nervous system. In PTSD patients, the persistent activation of microglia is considered a key mechanism for the exacerbation of inflammatory responses. Moreover, peripheral inflammatory signals can cross the blood–brain barrier to act on the central nervous system, further activating microglia while impairing their ability to maintain neural homeostasis (Butovsky and Weiner 2018).

Animal model studies provide evidence for this mechanism. For example, in mouse models of PTSD, excessive microglial branching has been closely associated with a significant reduction in neuronal dendritic spines (Smith et al. 2019). Furthermore, targeting and inhibiting the abnormal activation of microglia has been proposed as a potential strategy for treating PTSD (Li et al. 2021). These findings suggest that excessive microglial activation may be one of the key pathological features of PTSD.

Recent studies based on positron emission tomography (PET) have further elucidated the role of neuroinflammation in PTSD. For example, [^18^F]‐FEPPA PET imaging has shown significantly elevated microglial activation levels in the hippocampus and frontal cortex of PTSD patients, which are correlated with symptom severity (Deri et al. 2021). In addition, PET studies using the monoamine oxidase‐B probe [^11^C]SL25.1188 have revealed a significant increase in the number of astrocytes in PTSD patients, providing further evidence supporting the presence of neuroinflammation (Gill et al. 2022).

However, some studies have reported contradictory findings. For instance, [^11^C]PBR28 PET imaging showed that in certain PTSD subgroups, microglial activation levels in the prefrontal–limbic system were negatively correlated with symptom severity. In addition, postmortem studies found reduced expression of microglia‐associated genes (such as TNFRSF14 and TSPOAP1) in certain subgroups of female PTSD patients (Bhatt et al. 2020). These results highlight the heterogeneity and individual variability in the pathological mechanisms of PTSD, suggesting the need for more refined study designs to explore the neuroinflammatory characteristics of different subtypes.

#### 2.2.2 Involvement of Peripheral Immune Cells

Outside the central nervous system, studies on the peripheral immune system have also revealed characteristics of immune imbalance in PTSD patients. Compared to trauma‐exposed healthy controls, PTSD patients exhibit significantly elevated counts of peripheral white blood cells, lymphocytes, T cells, and CD4+ cells (Boscarino and Chang 1999). Among these, the proportions of Th1 and Th17 cells are increased, while regulatory T cells are reduced. This imbalance in immune cells is closely associated with elevated levels of pro‐inflammatory factors such as IFN‐γ and IL‐17 (Zhou et al. 2014). Other studies have reported a decreased CD4+/CD8+ T cell ratio in PTSD patients, accompanied by an increase in CD8+ effector T cells and naive T cells (Aiello et al. 2016). These findings indicate a pronounced pro‐inflammatory immune imbalance in the peripheral immune system of PTSD patients.

This may be because, under certain pathological conditions, peripheral immune cells (such as macrophages and T cells) can cross the blood–brain barrier and enter the brain parenchyma, leading to tissue damage. Under normal circumstances, peripheral immune cells present in the CSF and meninges do not typically invade brain tissue. Therefore, this could be an important direction for future research.

Based on current evidence, microglia play a central role in the neuroinflammatory response. By interacting with astrocytes, they exacerbate local inflammation while also interacting with pro‐inflammatory signals from the peripheral immune system. Together, these processes drive the neuropathological progression of PTSD. Future research should focus on the precise regulatory mechanisms of microglia, particularly their differential roles across various PTSD subtypes. Further exploration of the specific pathways of interaction between peripheral and central immune cells, as well as their effects on blood–brain barrier permeability, could help elucidate the pathophysiological mechanisms of PTSD. Microglial marker detection based on PET imaging may provide novel tools for PTSD subtyping and treatment monitoring, while immune therapies targeting the Th1/Th17 balance could offer new directions for personalized interventions.

### 2.3 Alterations in PTSD‐Related Immune Genes

#### 2.3.1 The Relationship between Genetic Variations and PTSD

At the genetic level, PTSD is closely associated with polymorphisms in various inflammation‐related genes. Studies have shown that variations in the major histocompatibility complex (MHC) gene family are significantly linked to abnormalities in neural and immune functions in PTSD patients (Yehuda et al. 2009). In particular, gene variants associated with HLA loci markedly increase the risk of developing PTSD, further highlighting the importance of genetic factors (Ahmed et al. 2024; Lori et al. 2024). In addition, recent evidence from large‐scale genome‐wide association studies (GWAS) has revealed bidirectional genetic correlations between PTSD and several immune‐related genes. This finding suggests that inflammation may not only act as a trigger for PTSD but also play a critical role in its pathological processes (Z. Wang et al. 2024; Zuo et al. 2023).

Studies on cytokine‐related genes have further revealed the complex relationship between gene polymorphisms and PTSD severity. For instance, the rs3091244 polymorphism of the CRP gene is closely associated with the alleviation of PTSD symptoms, while the rs1205 and rs2794520 polymorphisms may exacerbate symptoms (Miller et al. 2018). Similarly, in populations of war veterans, the rs1800629 polymorphism of the TNF gene is significantly correlated with the severity of PTSD symptoms (Bruenig et al. 2017). These findings provide critical insights into how gene polymorphisms contribute to the heterogeneity of PTSD symptoms.

#### 2.3.2 The Role of Epigenetic Modifications

Epigenetic studies have also shed light on the regulatory role of immune‐related genes in the development of PTSD. For example, increased methylation levels in the promoter region of the IL‐18 gene are significantly associated with the onset of PTSD in soldiers after military deployment (Rusiecki et al. 2013). In chronic PTSD patients, the expression of the IL‐16 gene is significantly downregulated, while the expression of the IL‐8 receptor is significantly upregulated, suggesting that specific epigenetic modifications of genes may sustain the inflammatory state (Zieker et al. 2007). Similarly, the methylation levels of the promoter regions of the IFN‐γ and IL‐12B genes are significantly elevated in PTSD patients, further supporting the critical role of epigenetic regulation in the pathological processes of PTSD (Bam et al. 2016). In addition, studies have found that methylation levels of the TLR1 and TLR3 genes are significantly reduced in PTSD patients, and these changes are negatively correlated with the severity of traumatic events (Uddin et al. 2010). These findings suggest that epigenetic modifications of immune‐related genes may profoundly influence the onset and progression of PTSD by regulating inflammatory responses.

These studies not only reveal the potential regulatory role of gene modifications in PTSD but also provide new pathways for exploring its molecular mechanisms. In the future, combined research integrating genomics and epigenetics will further clarify the specific mechanisms of immune‐related genes in PTSD. In particular, personalized diagnostic and therapeutic strategies based on specific genetic variations or epigenetic modifications—such as genetic screening to identify high‐risk individuals or the development of targeted therapies—hold promise for significantly improving early diagnosis and intervention outcomes for PTSD. This precision medicine approach not only provides a theoretical basis for the treatment and rehabilitation of PTSD but also lays the foundation for the clinical practice of personalized medicine.

## 3 Possible Mechanisms of Inflammation and Immune Dysregulation in PTSD

The mechanisms of inflammation and immune dysregulation in PTSD represent a complex interplay of multiple factors, involving interactions between the central and peripheral immune systems as well as abnormalities in neuroendocrine regulation. Inflammation plays a critical role in the onset and progression of PTSD through multiple pathways.

### 3.1 Central Nervous System Inflammation and Immune Dysfunction

The role of inflammatory factors in PTSD mainly involves the activation of glial cells, which alters synaptic plasticity and interferes with normal learning and memory processes. Studies have shown that the activation levels of microglia and astrocytes are significantly elevated in PTSD patients. These activated neuroimmune cells release pro‐inflammatory factors (such as NF‐κB) and other inflammatory mediators, leading to neuronal damage and cognitive dysfunction. This inflammation is primarily concentrated in brain regions such as the prefrontal cortex, amygdala, and hippocampus, respectively affecting emotional regulation, memory consolidation, and fear extinction.

### 3.2 Interaction Between Peripheral Immune System and Inflammatory Cytokines

Abnormal activation of the peripheral immune system also plays a significant role in the pathophysiological mechanisms of PTSD. The pro‐inflammatory state of peripheral monocytes disrupts the integrity of the blood–brain barrier, allowing inflammatory factors to enter the central nervous system, thereby creating a vicious cycle of central and peripheral inflammation. Moreover, chronic stress significantly enhances the expression of peripheral pro‐inflammatory factors, making the impact of peripheral immune activation on central inflammation more persistent and widespread.

### 3.3 Chronic Stress and Dysregulation of the HPA Axis

Chronic stress is a key driver of the inflammatory mechanisms in PTSD, significantly promoting the persistence and systemic expansion of inflammation. Prolonged stress leads to elevated levels of adrenaline and noradrenaline, which not only enhance the expression of peripheral pro‐inflammatory factors but also affect immune system regulation through dysfunction of the hypothalamic–pituitary–adrenal (HPA) axis. The HPA axis is a core endocrine pathway for responding to stress and regulating immune responses. In PTSD patients, HPA axis dysfunction is significantly observed, characterized by glucocorticoid resistance, which weakens the negative feedback regulation of inflammation, leading to elevated levels of pro‐inflammatory cytokines (such as TNF‐α and IL‐6). Studies have found abnormal cortisol secretion in PTSD patients, which further hinders the timely resolution of inflammation, maintaining a chronic inflammatory state. Furthermore, HPA axis dysregulation exacerbates central nervous system inflammation, particularly through the activation of microglia, releasing large amounts of pro‐inflammatory mediators, which further impair the function of the prefrontal cortex and hippocampus, worsening core PTSD symptoms. The abnormal HPA axis may aggravate the dysregulation between the central and peripheral immune systems, further driving the pathological progression of PTSD.

### 3.4 Abnormalities in Genetic and Epigenetic Regulation

Genetic and epigenetic alterations provide a foundational basis for the inflammatory state of PTSD. Variations in inflammation‐related gene polymorphisms and changes in their methylation levels may exacerbate the production of pro‐inflammatory factors, accelerating the onset and progression of PTSD at the molecular level. These genetic changes not only influence the expression of inflammatory factors but also regulate the central nervous system's response to traumatic stress, offering a multi‐layered pathological explanation for PTSD.

### 3.5 Dysfunction of the Autonomic Nervous System

Dysregulation of the autonomic nervous system (ANS) is another important pathological feature of PTSD. Studies have shown that PTSD patients often exhibit prolonged hyperactivity of the sympathetic nervous system (SNS) and significant suppression of parasympathetic nervous system (PNS) activity (Dunlop and Wong 2019). SNS hyperactivity leads to increased secretion of catecholamines, such as adrenaline and norepinephrine (Tan et al. 2011), which promotes the activation of the NF‐κB signaling pathway, thereby exacerbating the expression of pro‐inflammatory factors such as IL‐1 and IL‐6 (Bierhaus et al. 2003). The reduction in PNS activity, characterized by decreased heart rate variability, indicates weakened vagal tone, further contributing to the development of a chronic inflammatory state (Bandelow et al. 2017; Lehrer and Gevirtz 2014; Tracey 2009).

### 3.6 Aseptic Inflammation and the Mechanism of DAMPs

In recent years, sterile inflammation has gained widespread attention as an important inflammatory mechanism. Sterile inflammation is driven by endogenous damage‐associated molecular patterns (DAMPs), leading to a pro‐inflammatory state, rather than being triggered by pathogenic microorganisms. The significant elevation of DAMPs molecules, such as heat shock proteins, HMGB1, and ATP, has been confirmed as an important trigger of the inflammatory response in PTSD patients. Furthermore, the activation of the NLRP3 inflammasome exacerbates the PTSD‐related inflammatory response by promoting the release of pro‐inflammatory factors. This mechanism provides an important framework for understanding the breadth and persistence of the inflammatory response in PTSD.

## 4 Potential Anti‐Inflammatory Treatment Strategies for PTSD

### 4.1 Anti‐Inflammatory Potential of Approved Drugs

Currently, the only FDA‐approved medications for the treatment of PTSD are selective serotonin reuptake inhibitors (SSRIs), specifically paroxetine and sertraline. However, despite extensive research highlighting the critical role of inflammation in the pathogenesis of PTSD, studies investigating anti‐inflammatory therapeutic strategies remain relatively limited. Some studies have found that SSRIs not only alleviate PTSD symptoms but also exhibit anti‐inflammatory properties. For example, in patients with chronic PTSD, SSRI treatment has been shown to significantly reduce serum levels of the pro‐inflammatory cytokine IL‐1β (Tucker et al. 2004).

### 4.2 The Potential of Novel Anti‐Inflammatory Drugs

In addition to SSRIs, other anti‐inflammatory drugs have also demonstrated potential efficacy. For example, angiotensin‐converting enzyme inhibitors (ACE inhibitors) and angiotensin receptor blockers (ARBs) have been shown to alleviate PTSD symptoms and reduce levels of the pro‐inflammatory cytokine IL‐1β in patients with chronic PTSD (Kortekaas et al. 2014; Nylocks et al. 2015). In animal studies, the non‐steroidal anti‐inflammatory drug (NSAID) ibuprofen significantly improved PTSD‐like behaviors in PTSD models by inhibiting the expression of pro‐inflammatory cytokines in the hippocampus (Kao et al. 2016). These findings suggest that anti‐inflammatory treatments may regulate the pathological processes of PTSD through multiple pathways, providing important directions for drug development.

### 4.3 Dietary Interventions and Gut Microbiome Regulation

The gut microbiota plays a key role in regulating the gut–brain axis and has become another research hotspot in anti‐inflammatory treatments for PTSD. Studies have shown that gut microbiota dysbiosis may exacerbate PTSD symptoms by promoting neuroinflammation and immune dysregulation. Supplementing with probiotics or optimizing dietary structures—such as increasing the intake of dietary fiber and anti‐inflammatory foods—can improve gut microbiota composition, reduce inflammation levels, and aid in the recovery of cognitive function (Pivac et al. 2023). These interventions hold significant potential for application in the comprehensive treatment of PTSD in the future.

## 5 Future Directions

In recent years, although numerous studies have revealed evidence of inflammation and immune dysregulation in PTSD patients, most of these findings are based on peripheral blood studies, and direct evidence of inflammatory changes in the brains of PTSD patients remains limited. The pathogenesis of PTSD involves complex changes in brain function and structure, but current technologies face significant challenges in precisely localizing inflammatory changes to PTSD‐related brain regions. Current research largely relies on animal models for mechanistic exploration; however, due to the relatively slow development of technologies and methodologies, the neurobiological links between inflammation and abnormalities in brain function, structure, and behavior have yet to be fully elucidated. This research gap urgently needs to be addressed through advancements in new technologies and multidisciplinary collaboration.

Although the specific mechanisms linking PTSD and inflammation remain unclear, existing evidence suggests that inflammation and immune dysregulation may play a critical role in the onset and progression of PTSD, opening new avenues for research in psychopathology. Current studies often rely on single approaches, such as measuring inflammatory factors, immune cells, or gene expression in blood or saliva, but lack a comprehensive and multidimensional research framework. Future research should aim to integrate multiple methods, such as combining measurements of immune factor levels in blood and saliva with genetic analyses and functional magnetic resonance imaging (fMRI), to build a more holistic research model. In particular, fMRI, as a non‐invasive technique, holds significant value in exploring structural and functional changes in PTSD‐related brain regions, such as the prefrontal cortex, amygdala, and hippocampus. When combined with immunological research, this approach could reveal deeper mechanisms underlying PTSD.

Further research should focus on the specific mechanisms underlying inflammatory changes in PTSD, particularly the interactions between immune dysregulation, inflammatory factors, and core PTSD symptoms (e.g., fear memory, emotional dysregulation, and cognitive impairment). This could not only uncover the pathological basis of PTSD but also provide a scientific rationale for anti‐inflammatory therapies and identify new therapeutic targets. For example, clarifying the impact of inflammation on the central nervous system, especially its mechanisms of action within specific brain regions, holds promise for advancing more precise intervention strategies and guiding drug development for PTSD patients. Moreover, such studies could accelerate the development of early diagnostic tools based on inflammatory biomarkers, improving the efficiency of identifying high‐risk PTSD populations.

In summary, the relationship between PTSD and inflammation is a complex, multidimensional, and multilayered issue, involving changes at the genetic, molecular, and cellular levels, as well as the dynamic processes of neuro–immune interaction. Future research should focus on the following directions: first, further integration of neuroimaging and immunology to establish a multimodal research framework; second, clarifying the temporal sequence and specific pathways of inflammatory changes in the pathogenesis of PTSD; and third, advancing the development of anti‐inflammatory and immunomodulatory therapies and incorporating them into the comprehensive treatment system for PTSD. These studies could not only deepen our understanding of the pathological mechanisms underlying PTSD but also provide a theoretical foundation for developing novel diagnostic and therapeutic strategies. For instance, anti‐inflammatory therapies and personalized immunomodulation strategies may become critical breakthroughs for alleviating PTSD symptoms while improving patients' long‐term quality of life and recovery outcomes.

## Author Contributions

**Luodong Yang**: conceptualization, investigation, visualization, writing – original draft. **Jiaying Lu**: data curation, methodology, writing – original draft. **Zhiqiang Zhao**: writing – review and editing. **Ziwei Zhang**: data curation, methodology. **Weiliang Yang**: writing – review and editing, supervision. **Guiqing Zhang**: funding acquisition, project administration, supervision, conceptualization.

## Ethics Statement

The authors have nothing to report.

## Conflicts of Interest

The authors declare no conflicts of interest.

## Peer Review

The peer review history for this article is available at https://publons.com/publon/10.1002/brb3.70633

## Data Availability

The authors have nothing to report.
